# Mitochondrial biogenesis and neural differentiation of human iPSC is modulated by idebenone in a developmental stage-dependent manner

**DOI:** 10.1007/s10522-017-9718-4

**Published:** 2017-06-22

**Authors:** J. Augustyniak, J. Lenart, M. Zychowicz, P. P. Stepien, L. Buzanska

**Affiliations:** 1 Stem Cell Bioengineering Unit, Mossakowski Medical Research Centre Polish Academy of Sciences, Warsaw, Poland; 2 Department of Neurochemistry, Mossakowski Medical Research Centre Polish Academy of Sciences, Warsaw, Poland; 3 Department of Genetics and Biotechnology, Faculty of Biology, University of Warsaw, Warsaw, Poland; 4 Institute of Biochemistry and Biophysics, Polish Academy of Sciences, Warsaw, Poland; 5 Centre of New Technologies, University of Warsaw, Warsaw, Poland

**Keywords:** Idebenone, hiPSC, Neural progenitors, Developmental neurotoxicity, Mitochondrial biogenesis, mtDNA copy number, Neuronal, Astrocytic differentiation

## Abstract

Idebenone, the synthetic analog of coenzyme Q10 can improve electron transport in mitochondria. Therefore, it is used in the treatment of Alzheimer’s disease and other cognitive impairments. However, the mechanism of its action on neurodevelopment is still to be elucidated. Here we demonstrate that the cellular response of human induced pluripotent stem cells (hiPSC) to idebenone depends on the stage of neural differentiation. When: neural stem cells (NSC), early neural progenitors (eNP) and advanced neural progenitors (NP) have been studied a significant stimulation of mitochondrial biogenesis was observed only at the eNP stage of development. This coexists with the enhancement of cell viability and increase in total cell number. In addition, we report novel idebenone properties in a possible regulation of neural stem cells fate decision: only eNP stage responded with up-regulation of both neuronal (*MAP2*), astrocytic (*GFAP*) markers, while at NSC and NP stages significant down-regulation of *MAP2* expression was observed, promoting astrocyte differentiation. Thus, idebenone targets specific stages of hiPSC differentiation and may influence the neural stem cell fate decision.

**Electronic supplementary material:**

The online version of this article (doi:10.1007/s10522-017-9718-4) contains supplementary material, which is available to authorized users.

## Introduction

Human induced pluripotent stem cells (hiPSC) can generate neural stem cells, as well as neural and glial progenitors (Choi et al. 2014). Differentiation of hiPSC into neural progenitors is associated with the metabolic switch from glycolysis to oxidative phosphorylation (OXPHOS) and is correlated with an increase in the number of mitochondria (Zheng et al. 2016).

Mitochondria play a central role in energy production, apoptosis, and redox homeostasis. Loss of mitochondria functionality is observed in aging and age related neurodegenerative disorders. Manipulation of the activity and number of mitochondria is an interesting therapeutic option for age-related neurodegenerative diseases (Reddy 2009, Luo et al. 2015). In the process of reprogramming (Takahashi et al. 2007) somatic cells are converted into induced pluripotent stem cells. Among many physiological changes, de-differentiation involves the restructuring of mitochondria (mitochondria number, morphology, activity, and mtDNA amount). During differentiation, which is a process opposed to reprogramming, mitochondria plays a leading role as well (Wanet et al. 2015). Thus, a participation of mitochondria in reprogramming and differentiation is not only important in therapy and regenerative medicine but is also crucial for understanding stem cell biology.

Idebenone, an analogue of CoQ10, was synthesized in Japan in the 1980′s (Kanabus et al. 2014). This drug was intended for use for the treatment of neurodegenerative disorders and diseases that exhibit mitochondrial etiology. Idebenone was tested with varying degrees of success in Friedreich’s ataxia (Parkinson et al. 2013), Leber hereditary optic neuropathy (LHON) (Klopstock et al. 2011), mitochondrial encephalomyopathy (MELAS) (Napolitano et al. 2000), Duchenne muscular dystrophy (DELOS) (Buyse et al. 2015), multiple sclerosis (Villoslada 2016), dementia (Bergamasco et al. 1994), and Alzheimer’s disease (Weyer et al. 1997). Idebenone activates electron transport chain in mitochondria and exerts strong neuroprotective effect both in vitro and in vivo (Murphy et al. 1990; Ratan et al. 1994, Miyamoto and Coyle 1990). One of the most important features of idebenone is its antioxidant capacity (Jaber and Polster 2015). Mechanisms implicated in neuroprotection and antioxidative properties involve stabilization of the BAX/Bcl-2 ratio (Kernt et al. 2013). Reduced by complex II form of idebenone protects mitochondria against lipid peroxidation (Suno and Nagaoka 1989).

Until now, to our knowledge, the impact of idebenone on neural development in the stem cells models, including hiPSC has not been studied. Idebenone was tested in iPSC-derived Friedreich ataxia cardiomyopathy model on the drug screening platform and was shown not to influence tested cells in contrary to deferiprone (the iron chelator), which attenuated disease phenotype (Lee et al. 2016).

In this study, we present results showing the response of neural stem/progenitor cells generated from hiPSC to idebenone exposition at three different stages of neural differentiation: neural stem cells (NSC); early neural progenitors (eNP) and neural progenitors (NP). We have investigated cell viability, ROS level, mitochondrial membrane potential and mitochondrial biogenesis (SDHA and COX-1 protein level, mtDNA copy number), changes in total cell number as well as the expression of *NRF1, TFAM, PPARGC1A, MAP2, GFAP* genes. We have shown that idebenone can positively influence viability and total cell number, significantly increase mitochondrial biogenesis and can change lineage specification during neural differentiation of hiPSC.

## Materials and methods

### Cell culture and idebenone exposition

Before the exposition to idebenone (Sigma-Aldrich) at concentrations of 0.5; 0.25; 0.125 μM; control NSC, eNP and NP were generated from human induced pluripotent stem cells (hiPSC) (The Gibco^®^ Human Episomal iPSC Line, Thermo Fisher Scientific), as described in Augustyniak et al. 2017. Briefly, for neural differentiation, we used protocol adapted from Yan et al. 2013, with some modifications. Culture media and reagents were purchased from Thermo Fisher Scientific. At the undifferentiated stage, hiPSC were grown on a 6-well plate on rh-Vitronectin in Essential 8 Medium. The medium was replaced every other day. At 80% hiPSC confluency, Essential E8 Medium was changed to the PSC neural induction medium. hiPSC were grown in PSC Neural Induction Medium for the next days. The neural stem cells, was obtained after six passages maintained on Matrigel (BD Matrigel™ Basement Membrane Matrix, Corning) in Neural Expansion Medium (neural induction supplement 1:50, Neurobasal, Advanced DMEM, 1:1). The second stage of differentiation (eNP) was obtained from NSC by transferring cells to neural differentiation medium type I: Neurobasal, DMEM/F12 [1:1], N2 supplement 1%, B27 supplement 1%, EGF (20 ng/ml), bFGF (20 ng/ml), and culturing them for next 14 days. The third stage of differentiation (NP) was obtained from eNP by culturing in differentiation medium type I without EGF and bFGF (neural differentiation medium type II). Before exposition to idebenone, all three cells populations were seeded at a density of 5 × 10^5^ cells/cm^2^ on 6-well, 24-well or 96-well (Nunc) plates covered with the solution of Matrigel:DMEM/F12 (1:30) in medium dedicated to NSC (neural expansion medium), eNP (differentiation medium type I), NP (differentiation medium type II). The next day the media were replaced by the fresh ones supplemented with idebenone at concentrations of control; 0.125; 0.25; 0.5 µM. The cells were incubated with idebenone for 5 days.

### Immunocytochemistry

NSC, eNP, and NP were characterized by immunofluorescence staining. Images were prepared in Laboratory of Advanced Microscopy Techniques, Mossakowski Medical Research Centre Polish Academy of Sciences using Confocal Laser Microscope LSM 510 (Zeiss). hiPSC- derived neural stem cells, early neural progenitors and late neural progenitors were seeded on coverslips covered with solution of Matrigel: DMEM/F12, (1:30 ratio) in a 24-well plate (5 × 10^5^ cells/cm^2^) in the medium dedicated to the stage of development. At 80% confluency cells were fixed with 4% of PFA (15 min). At the next steps, cells were washed with PBS and 0.1% Triton X-100 was used for cells permeabilization. Before primary antibodies (Supplementary Table 1) were added, blocking solution of 10% goat serum was applied for 1 h and cells were incubated with primary antibodies for 24 h. After this time secondary antibodies (Supplementary Table 1) were added and incubated for 1 h in the dark. Nuclei were contrast gained with Hoechst 33258 (Sigma-Aldrich).

### Alamar Blue viability assay

After 5 days of exposure to idebenone at doses of 0–0.5 µM, the Alamar blue viability assay (Sigma-Aldrich) was performed. Fluorescence of resorufin was read at wavelengths: 544 nm (excitation) and 590 nm (emission) 3 h after adding reagent to the culture medium (1:10). The results are shown as the ratio (%) of the fluorescence intensity of test samples to the control (untreated) samples measured by Fluoroscan Ascent (FL, Labsystems) plate reader. Data presented on the graphs are normalized to cell number which was obtained by Janus Green (Abcam) staining performed according to the manufacturer’s protocol.

### ROS level detection

After 5 days of exposition of NSC, eNP, and NP to idebenone, ROS level was measured by DCFH-DA (dichloro-dihydro-fluorescein diacetate, Sigma-Aldrich) assay. Cells were incubated with DCFH-DA reagent (1 µM) for 3 h. After this time fluorescent DCF(2′,7′-dichlorofluorescin) was detected by a plate reader Fluoroscan Ascent (FL, Labsystems) at wavelengths: 485 nm (excitation)—538 nm (emission). The results are shown as the ratio (%) of the fluorescence intensity of test samples to the untreated control. Normalization of ROS level results to cell number was obtained using Janus Green (Abcam) staining.

### Mitochondrial membrane potential determination

After 5 days of exposition to idebenone, NSC, eNP and NP mitochondrial membrane potential was measured using fluorochrome Mitotracker^®^ Red CMXRos (Thermo Fisher Scientific) detected at the wavelength: 544 nm (excitation) 590 nm (emission) on a plate reader. The measurements were performed 4 h after the 50 nM solution of MitoTracker Red CMXRos was added to the culture medium. The results are shown as the ratio (%) of the fluorescence intensity of the test sample to the untreated control, after normalization of data to the total cell number with Janus Green staining according to manufacturer’s protocol.

### SDHA and COX-1 protein level determination

Cells at the three stages of neural differentiation were seeded separately on a 96-well plate covered with Matrigel solution (1:30). Idebenone was added to cells for 5 days. After this time the levels of two mitochondrial proteins was measured with MitoBiogenesis In-Cell ELISA Colorimetric kit (Abcam), according to the manufacturer’s instructions, on cells fixed with 4% PFA. SDHA, mt-COX-1 proteins level was obtained on a Fluostar plate reader OMEGA (BMG Labtech). After washing with PBS, cells were incubated for 30 min in 1X Permeabilization Buffer. Prior to the addition of primary antibodies (anti-SDHA and anti-COX-1), the cells were incubated in 2X Blocking Buffer for 2 h followed by a mix of secondary antibodies conjugated with enzymes: (1) alkaline phosphatase and (2) horseradish peroxidase for 60 min. After this time substrate for alkaline phosphatase was added and absorbance was measured at OD 405 nm wavelength, then the substrate for horseradish peroxidase was added, and absorbance was measured at OD 600 nm wavelength. Changes in SDHA and COX-1 levels were normalized to total cell number measured according to manufacturer’s protocol using Janus Green (Abcam) staining method. SDHA and COX-1proteins levels were shown independently as the absorbance ratio (%) of cell samples treated by idebenone to the untreated control.

### Total cell number determination

After 5 days of incubation with idebenone, cells were fixed with 4% PFA (RT). 1X Janus green reagent (Abcam) was added for the 5 min. After this time cells were washed five times with PBS, then 0.5 M HCl (10 min in RT) was added. Absorbance used to determine total cell number was measured on Fluostar plate reader OMEGA (BMG Labtech) at a wavelength: OD 595 nm. Total cell number was calculated from the standard curve and presented as the % of control cells untreated with idebenone.

### Gene expression analysis

#### DNA and RNA isolation

Total RNA and total DNA was extracted from NSC, eNP, NP samples with ZR-*Duet™* DNA/RNA Mini-Prep Kit (Zymo Research) according to the manufacturers’ protocols. Before reverse transcription reaction, total RNA was purified with Clean-Up RNA Concentrator kit (A&A Biotechnology, Gdynia, Poland), and RNA integrity on 2% agarose gels was validated. RNA and DNA concentration was measured using NanoDrop ND-1000 (Thermo Fisher Scientific, Waltham, USA). The purity of nucleic acids was assessed by calculating the 260/280 absorbance ratio.

#### RT-qPCR

**Fig. 1:**
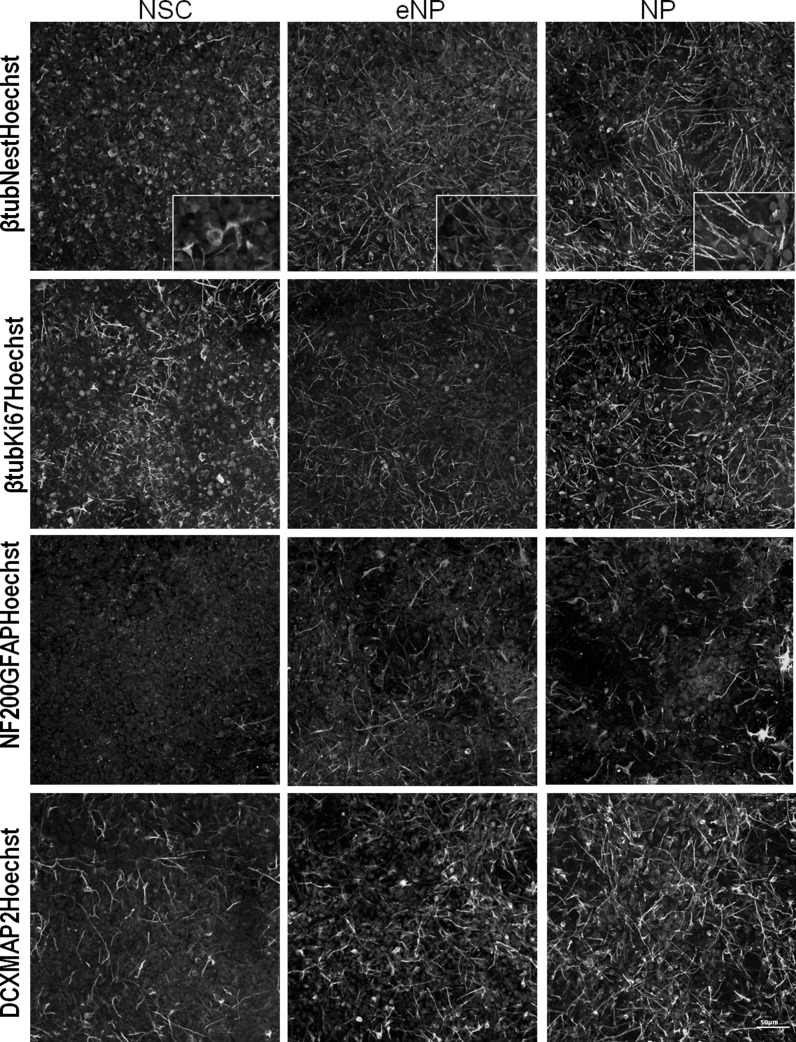
Immunocytochemical confirmation of: neural stem cells (NSC), early neural progenitors (eNP) and neural progenitors (NP). NSC culture exhibit dense and packed morphology, expressing the high level of early neural marker Nestin, proliferation marker Ki67 and early neuronal marker Doublecortin (DCX). During differentiation process, the morphology of cells changed to more elongated, branched, with decreasing Ki67 expression and increasing more advanced neuronal (β-TUBULIN3, MAP2, NF200) and astrocytic (GFAP) markers. *Scale bar* 50 µm

cDNAs to RT-qPCR reaction were obtained by High-Capacity RNA-to-cDNA™ Kit, (Thermo Fisher Scientific) on CivivCycler Thermocycler (Biotech INC). Gene expression analysis was evaluated with 1 μl (10 ng) cDNA template in 25 μl reaction mixture containing 12.5 μl iTaq™ Universal SYBR^®^ Green supermix (Bio-rad) and 0.25 μM/μl each primer (Supplementary Table 2). RT-qPCR was performed in the following steps: initial denaturation step at 95 °C for 3 min, 45 cycles of denaturation at 95 °C for 10 s, and annealing/extension at 60 °C for 1 min. Samples were tested in four replicates. For each stage of development (NSC, eNP, NP) reference gene was selected in NormFinder software as shown in Fig. 1. As a reference genes for a NSC stage were used: TBP, UBC, GAPDH, EID2, RABEP2, ZNF224B, PHB, CCNG1, CAPN10, EEF1A1, TUBB2; for eNP: RPLP0; for NP: EID2, RPLP0, UBC. Potential reference genes were described by Synnergren et al. 2007; Coulson et al. 2008; She et al. 2009; Eisenberg and Levanon 2013; Vossaert et al. 2013. GeneEx 6.1 software (MultiD Analyses AB, Göteborg, Sweden) was used to analyze the data by Pffafl method (Pfaffl, 2001) (the quantification cycle (Cq) values and the baseline settings automatically calculated by the qPCR instrument software) from LightCycler^®^ 96 Software (Roche Diagnostics GmbH, Mannheim, Germany).

#### qPCR

The mtDNA copy numbers were calculated as (1) *mt*-*ND1*/*SCLO2B1* ratio and (2) *mt*-*ND5*/*SERPINA1* ratio (Yu et al., 2012) on the quantification cycle (Cq) values and the baseline settings automatically calculated by the qPCR instrument software (LightCycler^®^ 96 Software, Roche Diagnostics GmbH, Mannheim, Germany). The qPCR was performed with 1 μl (10 ng) DNA template in 25 μl reaction mixture containing 12.5 μl iTaq™ Universal SYBR^®^ Green Supermix (Bio-rad) and 0.25 μM/μl each primer (Supplementary Table 3). PCR conditions were as folows: hot start at 95 °C for 3 min followed by 45 cycles of denaturation at 95 °C for 10 s, annealing at 60 °C for 30 s, and extension at 72 °C for 30 s.

### Statistical analysis

GraphPad Prism5.0. was used to perform statistical analysis. Kolmogorov-Smirnov was used as a normality test. After checking whether the distribution is normal test groups were compared with (1) the t-student test, (2) One-way ANOVA followed by Tukey’s Multiple Comparison Test (3) two-way ANOVA followed by Bonferroni Multiple Comparison Test; (*) p < 0.05 ; (**) p < 0.01; (***) p < 0.001; (****) p < 0.0001; for comparison inside the group; 2) (#) p < 0.05; (##) p < 0.01; (###) p < 0.001; (####) p < 0.0001 for comparison between group. Results represent three independent experiments, each in at least four replicates. Results presented at the graphs were shown as mean with standard error of measurement (SEM). Data in the text were present as mean with standard deviation (SD).

## Results

### Neural differentiation of hiPSC

NSC, eNP and NP were obtained from hiPSC as described before (Augustyniak et al. 2017). The panel of immunocytochemical images (Fig. 1) shows the staining of specific neural markers in cell the populations tested in this report. They included: Nestin (NEST)—the marker of the early stage of neural commitment; β-tubulin III (βTUB III), Doublecortin (DCX), Neurofilament 200 (NF200) and Microtubule Associated Protein 2 (MAP2)—the markers of neuronal differentiation; Glial Fibrillary Acidic Protein (GFAP)—the astrocytic marker and Ki67—indicating proliferating cells. From NSC through eNP to NP stage, lineage-related cells acquire more neuronal phenotype, as revealed by the enhanced expression of βTUB III, NF200, and MAP2, while decreasing level of NEST. DCX level was detected on similar level at all tested stages of development. Astroglial marker—GFAP is gradually increasing during differentiation, while the proliferation marker Ki67 gradually disappears, being the most abundant in NSC stage of development. The advancement in neuronal differentiation, as revealed by the expression of typical markers on the protein level, is shown to be correlated with the change in morphology from rounded to elongated cells with long protrusions (insets in Fig. 1). Detailed phenotype characterization of NSC, eNP and NP on mRNA level by RNA-seq method was presented previously by our group (Augustyniak et al. 2017).

### Influence of idebenone on cells viability

We observed an increase in viability at NSC at all tested doses. Only in NP stage, at dose 0.25 µM cell viability was lowered significantly [87.75% (±3.93)], but at 0.5 µM small, no significant increase was observed. The largest increase of viability was detected at eNP stage [138.19% (±10.78)] at the highest dose 0.5 µM of idebenone. The increase in cells viability was also high and significant for NSC for doses 0.25 µM [128% (±6.31) and 0.5 µM (130% (±4.40)].

**Fig. 2:**
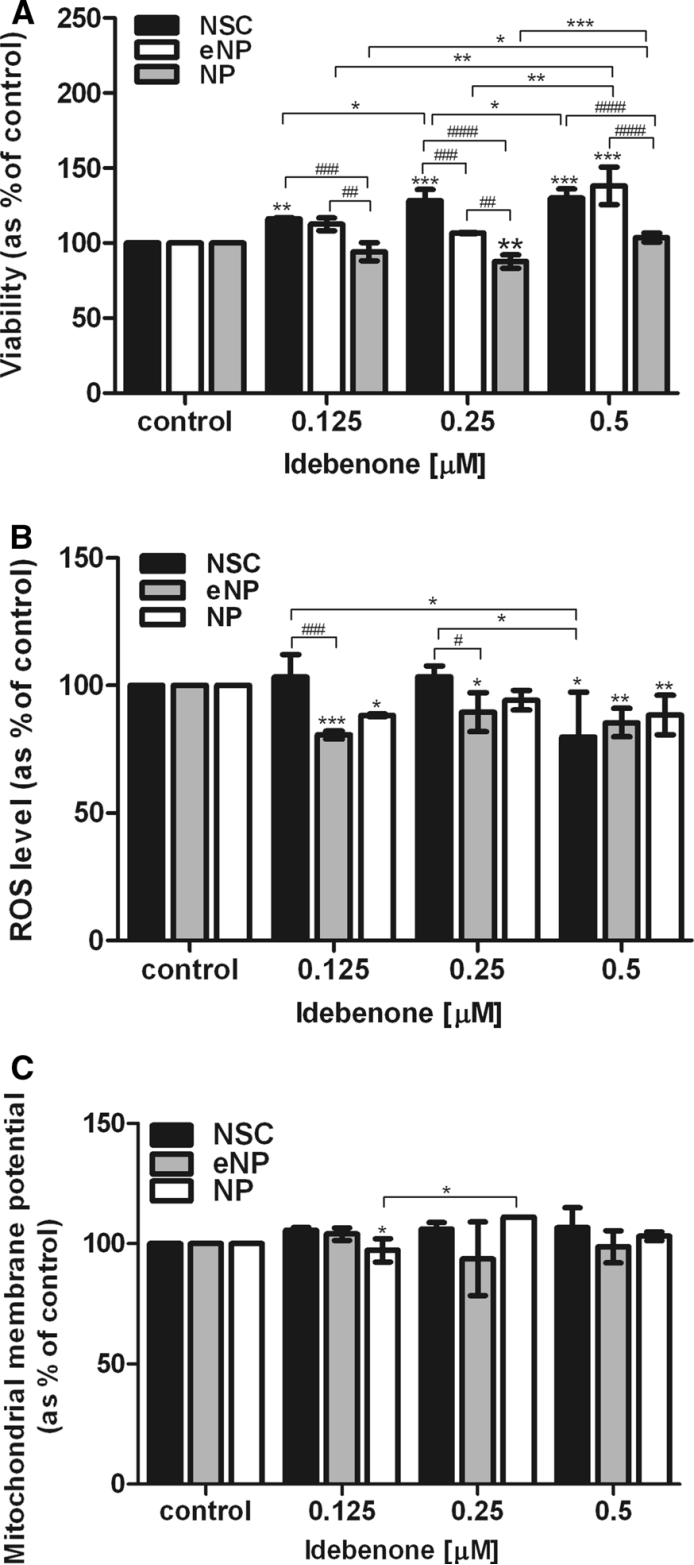
The cells at three different stages of neural differentiation: NSC, eNP and NP after 5 days of exposure to the different concentrations of idebenone were tested for (a) viability measured by Alamar Blue assay; (b) ROS level measured by DCFH-DA assay; (c) the mitochondrial membrane potential measured by MitoTracker Red CMXRos staining. After normalization to total cell number results are shown the mean (SEM) of fluorescencee intensity (%) of treated group versus control. *Brackets* show statistical significance between samples versus control (one way ANOVA, Tukey’s post-test): *p < 0.05; **p < 0.01; ***p < 0.001; ****p < 0.0001 and comparison between groups (two way ANOVA, Bonferroni post-test): ^#^p < 0.05; ^##^p < 0.01; ^###^p < 0.001; ^####^p < 0.0001

When the tested populations were compared at the highest dose of idebenone (0.5 µM), the differences between NSC [130% (±4.40)] versus NP [103.81% (±2.45)] and eNP [138.19% (±10.78)] versus NP [103.81% (±2.35)], were highly significant (####; p < 0.0001). The results are shown in Fig. 2a.

### Antioxidant properties of idebenone

Antioxidant properties of idebenone, revealed by reactive oxygen species (ROS) detection as the decrease in ROS level, have been observed at eNP and NP stages of neural differentiation. The most effective antioxidant capacity was recorded at the highest tested dose 0.25 µM for NSC [79.77%(±14.47)] and 0.5 μM for eNP [85.45% (±4.81)]; NP [88.44% (±7.09)]. A significant difference between compared populations (NSC vs. eNP vs. NP) at the dose 0.5 μM was not notified, as presented in Fig. 2b.

### Impact of idebenone on mitochondrial membrane potential

There were no significant changes in the accumulation of fluorescent dye between stages of differentiation at any tested dose of idebenone (Fig. 2c). Mitochondrial membrane potential was constant and for the cells treated with 0.5 μM idebenone revealed the level: NSC 106.66% (±18.54), eNP 98.76% (±13.38) and NP 103.16% (±3.64) of the control (100%) (Fig. 2c).

### Effect of idebenone on SDHA and COX-1 expression

**Fig. 3:**
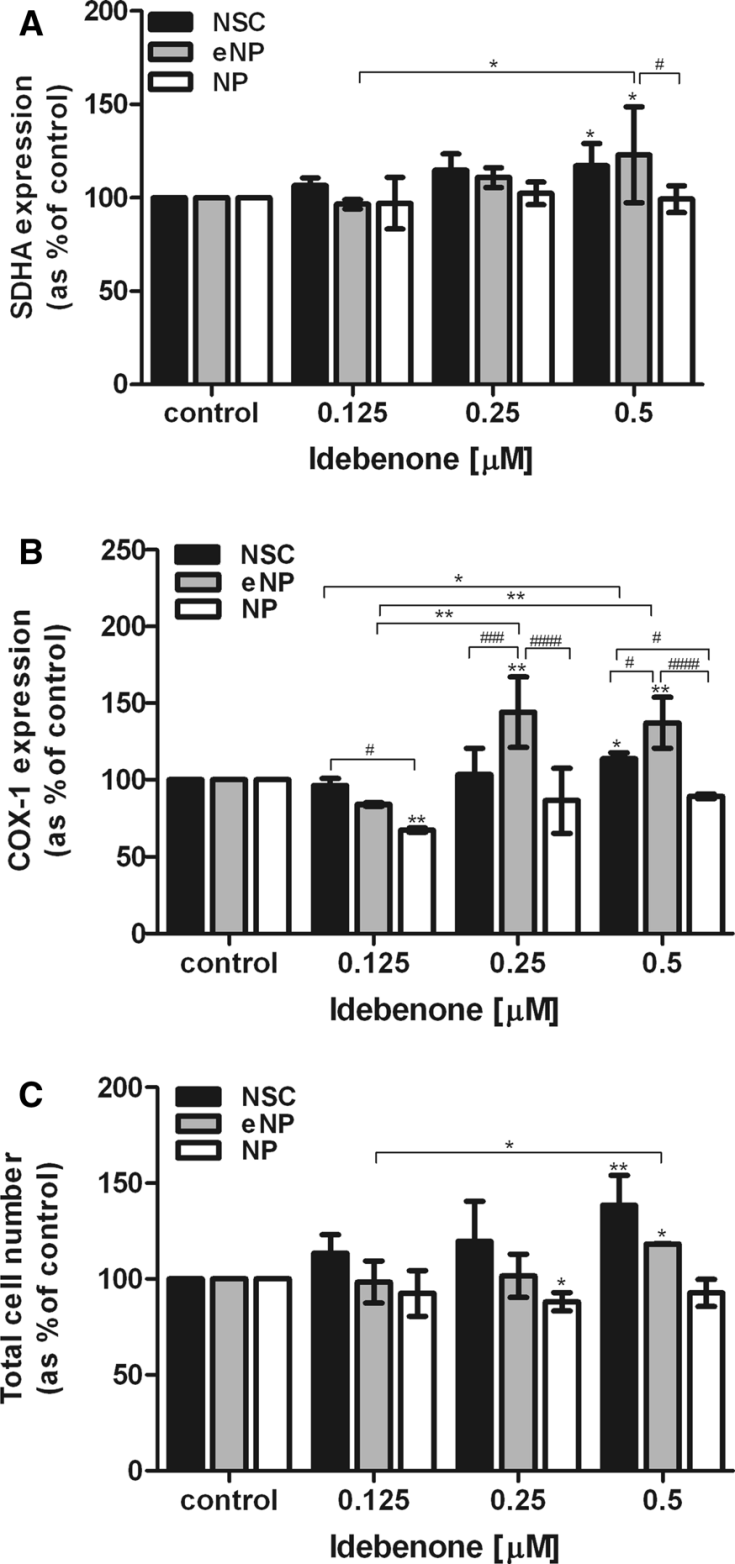
In the developmental stages: NSC, eNP and NP after 5 days exposure to the different concentrations of idebenone. **a** The Succinate Dehydrogenase Complex Flavoprotein Subunit A (SDHA) level. **b** The Cyclooxygenase (COX-1) level; **c**) Total cell number obtained from standard curve (Janus green staining) was measured. Results are shown as mean (SEM) relative percent (%) of absorbance versus control. The SDHA and COX-1 protein level were normalized to cell number with Janus green staining kit. Brackets show statistical significance between samples versus control (one way ANOVA, Tukey’s post-test): *p < 0.05; **p < 0.01; ***p < 0.001; ****p < 0.0001; and comparison between groups (two way ANOVA, Bonferroni post-test): ^#^p < 0.05; ^##^p < 0.01; ^###^p < 0.001; ^####^p < 0.0001

The SDHA (Flavoprotein (FP) subunit A of succinate dehydrogenase) protein level shown a significant effect for the highest dose of idebenone 0.5 µM: [123.12% (±20.94)] in the eNP and [117.04% (±10.79)] in the NSC stage. We did not observe any statistically significant difference of SDHA protein level between: NSC versus eNP contrary to eNP versus NP (#, p < 0.05) (Fig. 3a).

Idebenone significantly increased COX-1 (cytochrome C oxidase I) protein level in the early neural progenitors at dose 0.25 µM [144.13% (± 16.26)]; (**, p < 0.01) and 0.5 µM [137.24% (±11.83)]; (**, p < 0.01). At the highest dose significant enhancement of COX-1 protein level was detected in NSC stage of development [113.90% (±3.01)]; (* p < 0.05). No significant changes between treated and untreated control were detected at stage of NP at the dose 0.25 μM and 0.5 μM after 5 days of exposition to idebenone. We noticed significant difference in response to idebenone between: NSC versus eNP (#, p < 0.05); eNP versus NP (####, p < 0.0001), and NSC versus NP (#, p < 0.05) in the highest dose (Fig. 3b).

### Influence of idebenone on the total cell number

Idebenone increased total cell number of NSC in all tested doses: 0.125 µM [113.64% (±8.17)]; 0.25 µM [119.75% (±18.59)]; 0.5 µM [138.57% (±12.69)], but only for the highest dose it was statistic significant. The statistically significant differences between treated cells and untreated control were shown at highest dose of 0.5 µM for NSC [138.57% (±12.69)] and eNP [118.29% (±0.11)]. At stage of late neural progenitor (NP) idebenone inhibited total cell number, about ~7–12% versus control samples at all tested doses, however this effect was found to be statistically significant only for 0.25 µM dose of idebenone [88.10% (±3.87)] (Fig. 3c).

### Effect of idebenone treatment on mitochondrial DNA content

**Fig. 4:**
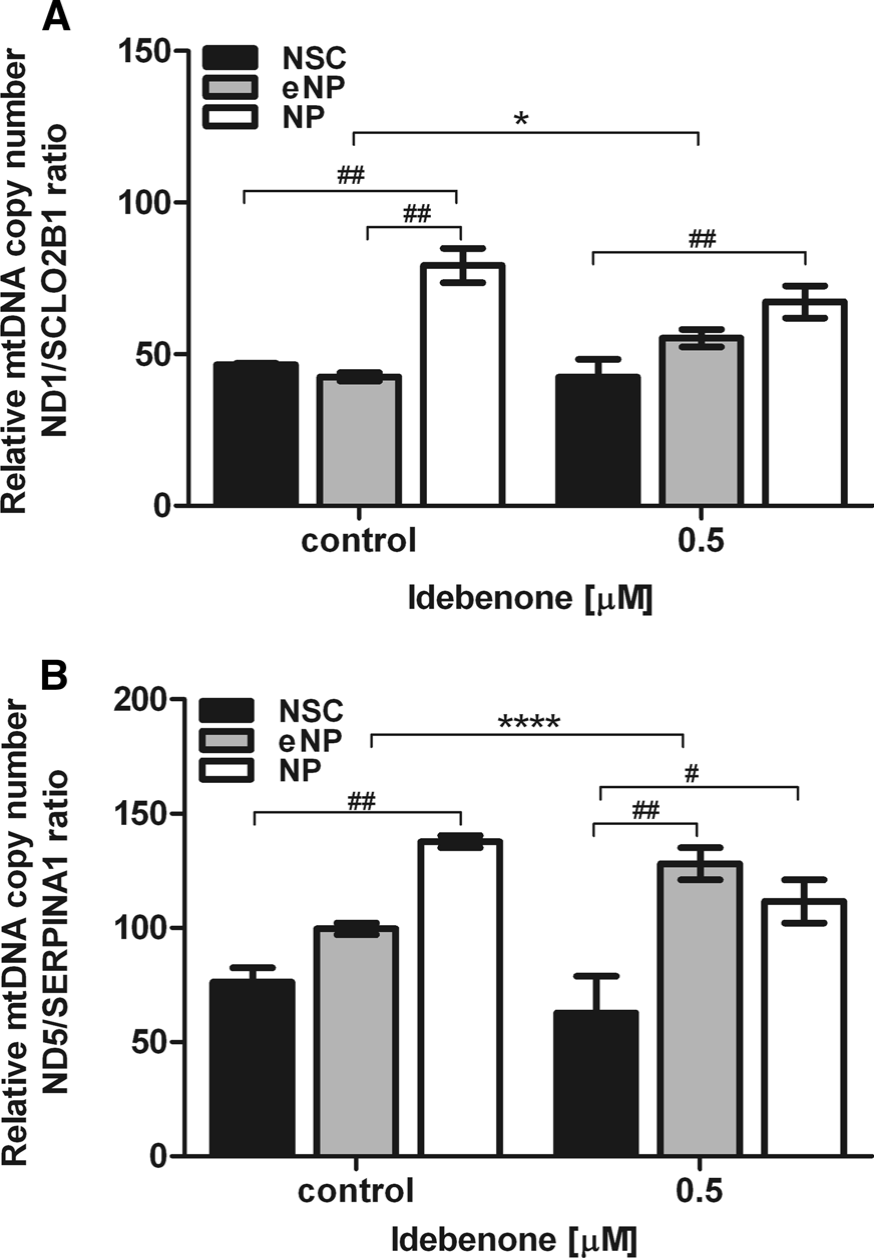
The relative mtDNA copy number estimated with qPCR measurement of *ND1, ND5, SLCO2B1* and *SERPINA1* level in NSC, eNP and NP after 5 days of exposure to the 0.5 µM idebenone: **a** *ND1/SLCO2B1* ratio and **b** *ND5*/*SERPINA1* ratio. Data are presented as mean (SEM). *Brackets* show statistical significance between samples versus control, (t-Student): *p < 0.05; **p < 0.01; ***p < 0.001; ****p < 0.0001 and comparison between groups (two way ANOVA, Bonferroni post-test): ^#^p < 0.05; ^##^p < 0.01; ^###^p < 0.001; ^####^p < 0.0001

Idebenone significantly increased ratio of *ND1/SCLO2B1* (*,p < 0.5) from 42.60 (±2.73) to 55.33 (±4.03) only at early neural progenitors stage (Fig. 4a). *ND1/SCLO2B1* ratio remains unchanged not significant in the two other stages (NSC and NP). Significant difference between tested groups was observed at untreated cells populations: NSC versus NP (##, p < 0.01) and eNP versus NP (##, p < 0.01). In cells treated by idebenone significant difference in response to stimulations was noted between NSC and NP (##, p < 0.01).

*ND5/SERPINA1* ratio analysis has shown significant increase at eNP stage (****, p < 0.0001) [128.00(±7.00)] as compared to untreated control [99.50(±2.50)]. At NSC and NP stages *ND5/SERPINA1* ratio was not changed at significant level (Fig. 4b). The significant difference between populations treated by idebenone was obtained for NSC [62.67(±22.87)] versus NP [111.50 (±9.50)] (#, p < 0.05) and NSC versus eNP [128.00 (±7.00)]. In untreated samples significant difference appeared only between NSC [76.25 (±11.03)] and NP [137.67 (±3.77)] (##, p < 0.01), as presented in Fig. 4b.

### Effect of idebenone on the relative gene expression involved in mitochondrial biogenesis and neural differentiation

**Fig. 5:**
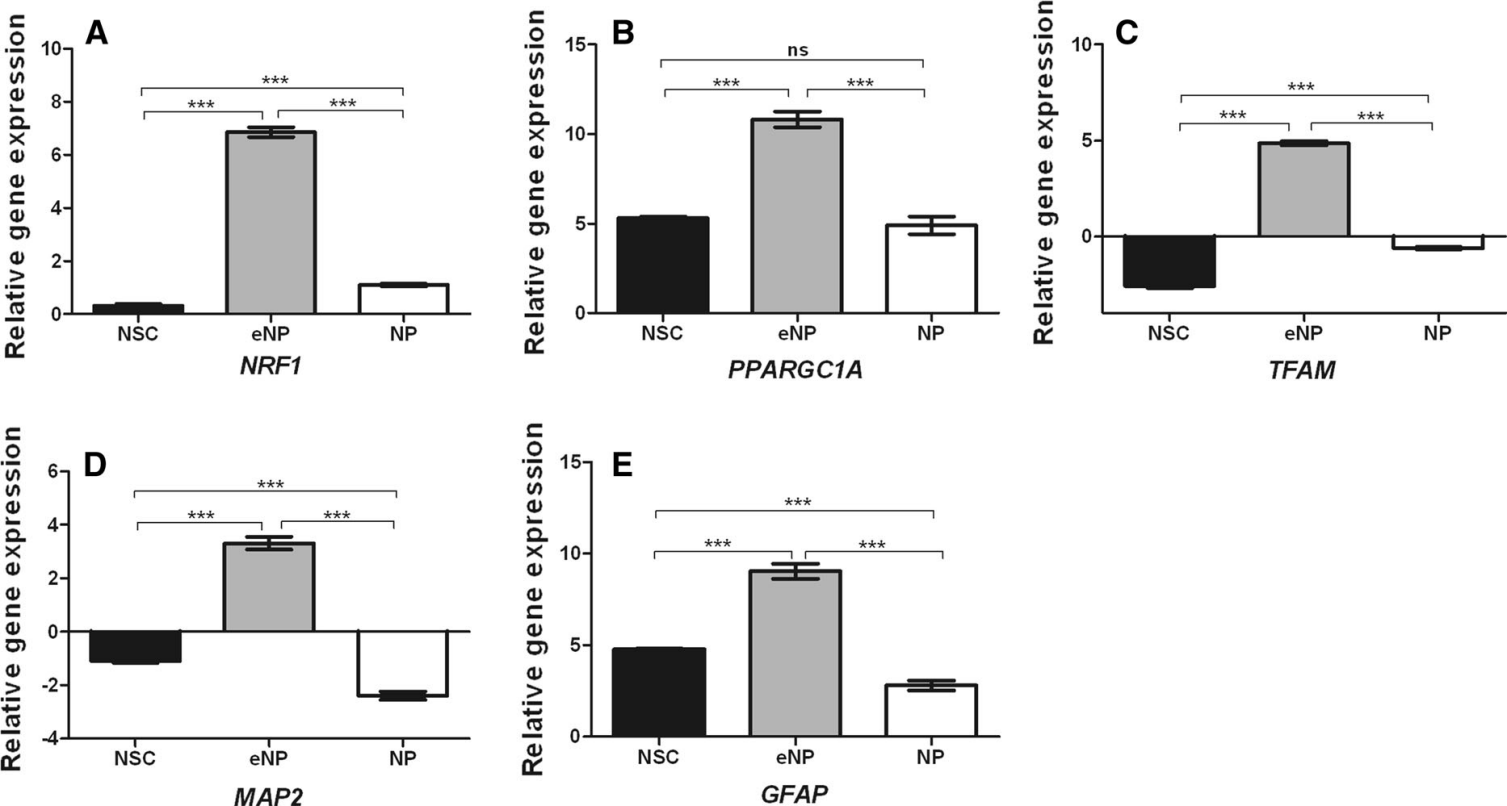
Real-time (RT-qPCR) evaluation of expression of genes involved in mitochondrial biogenesis and neural differentiation at the NSC, eNP, NP developmental stages. Relative expression of **a** *NRF1*, **b** *PPARGC1A*, **c** *TFAM*, **d** *GFAP*, **e** *MAP2* was measured after 5 days of cell treatment with 0.5 µM idebenone. Data are presented as the mean (SEM) from three independent experiments, each in four replicates. Brackets show statistical significance comparison between groups (one way ANOVA, Tukey’s post-test): *p < 0.05; **p < 0.01; ***p < 0.001; ****p < 0.0001

RT-qPCR analysis showed that *NRF1* mRNA expression level after exposure to idebenone at the stage of the early neural progenitors (eNP) increased about ~sevenfold [6.86 (±0.416)]. The increase in expression has also been observed in NSC and NP stages but it was much lower and ranged [0.311 (±0.156)] and [1.092 (±0.113)], respectively. We have also observed statistically significant differences in *NRF1* mRNA expression level between every tested developmental stage (***, p < 0.001) (Fig. 5a).

Idebenone enhanced *PPARGC1A* gene expression in all tested cell populations. The best efficiency in enhancing of *PPARGC1A* level is observed at the eNP stage, where relative gene expression was elevated about ~11-fold [10.824 (±0.998)]. Idebenone stimulated about ~5 times increase of *PPARGC1A* expression in NSC [5.304 (±0.164)] and NP [4.903 (±1.098)] stage of development. Relative gene expression in NSC versus eNP and eNP versus NP differed significantly (***, p < 0.001) while NSC versus NP population was not significantly different (Fig. 5b).

Idebenone raises the expression of *TFAM* gene about ~fivefold [4.865 (±0.233)], in early neural progenitors. At the developmental stages NSC and NP expression of *TFAM* was reduced about three times [−2.578 (±0.252)] and one time [−0.612(±0.131)], respectively (Fig. 5c). Difference in the *TFAM* gene expression was statistic significantly between all compared cells populations (***, p < 0.001).

Idebenone enhanced the *MAP2* relative gene expression only at the stage of early neural progenitors ~threefold increase [3.313 (±0.537) of expression]. At the stage of neural stem cells and neural progenitors (NP) the drug has the opposite effect: in NSC expression of *MAP2* decreased about ~onefold [−1.088 (±0.168)] and for NP about ~twofold [−2.391 (±0.347)]. The observed changes were significantly different between all stage of differentiation (***, p < 0.001) (Fig. 5d).

Idebenone was found to up-regulate about ~ninefold [9.040 (±0.925)] the *GFAP* gene expression level in early neural progenitors (eNP). Up regulation has been observed also at NSC ~fivefold [4.765 (±0.124)] and NP ~threefold [2.805 (±0.591)] stages of differentiation. Significant differences were recorded in all tested populations: NSC versus eNP versus NP (***, p < 0.001) (Fig. 5e).

## Discussion

In this paper, we demonstrate the cellular and molecular response of hiPSC-derived cell populations to the synthetic analog of coenzyme Q10, idebenone during early stages of neural development. To the best of our knowledge, the effect of idebenone has not been studies in neural stem cells so far. We assumed that due to the positive effect of idebenone on electron transport in mitochondria (Haefeli et al. 2011; Erb et al. 2012) and metabolic changes characteristic of stem cell development, such treatment may influence the mitochondrial biogenesis and neural differentiation pathway of hiPSC-derived neural stem cells in a stage-dependent manner: neural stem cells, early neural progenitors and neural progenitors.

Neural stem cells are multipotent cells characterized by self-renewal, the ability to proliferate without a limit and the capacity to produce neural progenitors which finally can be differentiated into neurons, astrocytes and oligodendrocytes (Clarke et al. 2000). Neural progenitors can be multipotent, bipotent or unipotent. They differ from NSC by limited capacity to self-renew, the restricted ability of neuronal and glial differentiation and in morphology what is revealed by elongated cell shape and the presence of protrusions extending from the cell body (Seaberg and van der Kooy 2003). During hiPSC differentiation cells gradually turn off naive pluripotency (Radzisheuskaya and Silva 2014) and acquire lineage markers shown as elevated expression of SOX2 and NESTIN (Verpelli et al. 2013) We have confirmed in our previous study (Augustyniak et al. 2017) the loss of pluripotency markers: *OCT4* in all tested populations and gradual silencing of *NANOG* with elevated expression of *SOX2* and *NESTIN* during neural commitment from hiPSC and gradual upregulation of the expression of *MAP2* and *GFAP* during further differentiation, which is also consistent with observations of other groups (Denham and Dottori 2011, Kwon et al. 2012, Verpelli et al. 2013). In the neural progenitors, which are more advanced in development, neurites are extended and the increase of the expression of MAP2 is observed (Denham and Dottori 2011). Nestin is a marker of neural differentiation, which can be detected at the earliest. Thus, the expression of early neural marker such as Nestin was shown after the induction of neural differentiation in the NSC at protein level (immunocytochemistry) and mRNA level (RT-PCR), with the tendency to diminish expression at the last NP stage. The expression of key proteins typical for neural development stages NSC, eNP and NP untreated with idebenone was tested at the all populations is presented in Fig. 1. It revealed gradual increase of the expression of βTUB III, DCX, NF200, MAP2 and GFAP along differentiation. The results obtained for tested markers confirmed data, which were previously obtained by our group on both protein (quantification of immunocytochemistry) and mRNA (quantitative analysis with RNA-seq) level (Augustyniak et al. 2017). The latter additionally proved, that presented cell populations significantly differ between each other and can be used as the independent stages of neural differentiation.

To elucidate whether idebenone can influence the neural differentiation process and content of mitochondria, and gene expression of key regulators of mitochondrial biogenesis have been investigated in the three above-mentioned cell populations. Thus, viability, total cell number, ROS level and mitochondrial membrane potential, expression of proteins (SDHA, COX-1) and genes (*NRF1, TFAM, PPARGC1A*), mtDNA copy number (revealed as *ND1/SCLO2B1* and *ND5/SERPINA1* ratio), important marker of mitochondrial biogenesis and the level of neural differentiation related markers (*MAP2* and *GFAP*) were evaluated upon the treatment with idebenone. We have observed that most effective was the highest concentration of idebenone tested by us (0.5 μM) therefore, the further comparison between NSC, eNP and NP cells was performed at the highest tested concentration of idebenone. The summary of the influence of idebenone (0.5 µM) on three different stages of neural differentiation is presented in Table 1.

**Table 1:** Summary of the influence of Idebenone (0.5 µM) at three different stages of neural differentiation (*, p<0.05; **, p<0.01, ***, p<0.001, ****, p<0.0001, ns-non-significant)

	NSC	ENP	NP
Viability	↑ (**)	↑ (**)	↑ (ns)
ROS level	↓ (*)	↓ (**)	↓ (**)
Mitochondrial membran potential	↑ (ns)	↓(ns)	↑ (ns)
Total cell number	↑ (**)	↑ (*)	↓ (ns)
Protein expresion			
SDHA	↑ (*)	↑ (*)	↓ (ns)
COX-1	↑ (*)	↑ (**)	↓ (ns)
mtDNA copy number			
*ND1/SCLO2B1ratio*	↓ (ns)	↑ (*)	↓ (ns)
*ND5/SERPINA1ratio*	↑ (ns)	↑ (****)	↓ (ns)
Gene expression (fold change)			
*NRF1*	↑ (0.31)	↑ (6.86)	↓(1.09)
*TFAM*	↓ (−2.58)	↑ (4.87)	↓ (−0.62)
*PPARGC1A*	↑ (5.30)	↑ (10.83)	↓ (4.90)
*MAP2*	↓ (−1.09)	↑ (3.31)	↓ (−2.39)
*GFAP*	↑ (4.77)	↑ (9.04)	↑ (2.805)

The population of NP did not respond significantly to the drug treatment for the most of the tested parameters (Table 1). In contrast to NSC and eNP populations, no significant changes were detected in cell viability (Fig. 2a), SDHA (Fig 3a), COX-1 protein level (Fig. 3b) and total cell number (Fig. 3c). The only exception was the significant reduction of the ROS level which is in agreement with previously reported function of idebenone as an antioxidant (Jaber and Polster 2015) and similar to the response of other tested populations (Fig. 2b).

The expression of proteins and genes implicated in the mitochondrial biogenesis was also tested. In our study we decided to measure the levels of two proteins: a major catalytic subunit of succinate-ubiquinone oxidoreductase (SDHA) a complex of the mitochondrial respiratory chain (Hirawake et al. 1994) (Fig. 3a) and mitochondrial complex IV: cytochrome c oxidase subunits (COX-1) (Anderson et al. 1981). The elevated level of SDHA (nuclear encoded) and COX-1 (mitochondrial encoded) is used as a marker of the one of the mitochondrial biogenesis marker since both of these proteins are localized and function in mitochondria. Thus, SDHA and COX-1 level were significantly elevated in NSC and eNP stages, while NP population was not affected.

The developmental stage dependent elevation of mitochondrial biogenesis after idebenone treatment was also confirmed by the analysis of mitochondrial DNA content. The relative DNA copy number was calculated from representative mitochondrial to nuclear genes ratio (*ND1/SCLO2B1* and *ND5/SERPINA1*). Idebenone induced significant increase of mtDNA copy number only in the stage of eNP, while NSC and NP populations did not respond significantly (Fig. 4a, b).

The genes that have been evaluated in our study: *NRF1*, *PPARGC1A* and TFAM are the key regulators of signaling pathways implicated in mitochondrial biogenesis (Kanabus et al. 2014). PGC1-alpha encoded by *PPARGC1A* is the major factor which by the cascade of nuclear-encoded hormone receptors, transcription factors, and transcriptional co-activators, including PPARs, estrogen-related receptors, thyroid hormone receptors, nuclear respiratory factors NRF1 and NRF2 and the transcription factors CREB and YY1 (Andreux et al. 2013; Dominy and Puigserver 2013; Kanabus et al. 2014) is able to increase the number of mitochondria. Several neurodegenerative diseases (Huntington´s Disease, Alzheimer´s Disease, and Parkinson´s Disease) are related to the impaired expression and function of PGC1 alpha. Signaling cascade regulated by PGC1 alpha is an attractive therapeutic target for mitochondrial-based diseases (Valero 2014; Kanabus et al. 2014). TFAM is a mitochondrial transcription factor 1 regulating the mitochondrial DNA replication and repair (Tiranti et al. 1995). Therefore in our research, we examined gene expression of *NRF1, PPAPGC1A, TFAM* and demonstrated by RT-qPCR analysis, that all these factors are significantly elevated only in eNP stage of development (Fig. 5a, b, c; Table 1). Up-regulation of *NRF1* and *PPARGC1A* and down-regulation of *TFAM* were shown in cells of NSC and NP stage of development.

The last but not least was the coexistence of the abovementioned changes with the neural fate commitment. For that purpose, the gene expression of *MAP2* and *GFAP*, a neuronal and astroglial marker, respectively, was investigated in the idebenone treated populations. While the *GFAP* was significantly upregulated in all tested populations, *MAP2* was shown to be repressed in NSC and NP populations and upregulated only at an eNP stage. What’s more, the upregulation of *GFAP* at eNP stage was twice as strong as in other tested stages (Fig. 5d, e; Table 1). Thus surprisingly, we observed that only at the stage of early progenitors idebenone could up-regulate both *MAP2* and *GFAP* gene expression, which suggests that a bidirectional induction of differentiation is possible only at this specific stage of differentiation. Furthermore, the specific for eNP upregulation of all tested genes involved in the mitochondrial biogenesis as well as significant upregulation of viability may suggest that eNP stage is the “developmental window of sensitivity” for the neuroprotective function of the idebenone. More research is needed to elucidate the effect of idebenone on mitochondrial biogenesis and stem cell fate decision during neural differentiation, however, based on the presented results we can strongly support the hypothesis that idebenone protective effect is developmental stage dependent and that future targeted treatment of the selected stage of neural development may exert better therapeutic effect.

## Electronic supplementary material

Below is the link to the electronic supplementary material.

Supplementary material 1 (DOCX 14 kb)

Supplementary material 2 (DOCX 17 kb)

Supplementary material 3 (DOCX 14 kb)
