# Supplementary material for: Mitochondrial biogenesis and neural differentiation of human iPSC is modulated by idebenone in a developmental stage-dependent manner
Source: Biogerontology. 2017 Jun 22;18(4):665–77. doi: 10.1007/s10522-017-9718-4 (PMC5514205; doi:10.1007/s10522-017-9718-4)
Supplement: Supplementary file 1 — Supplementary material 1 (DOCX 14 kb) [file 10522_2017_9718_MOESM1_ESM.docx]

Suppl.Tab.1. Antibodies applied for the immunocytochemistry staining of NSC, eNP, NP

| *Primary antibodies* | | | *Secondary antibodies* | | |
| --- | --- | --- | --- | --- | --- |
| *Name* | *Company that*  *produces antibodies* | *Dilution* | *Name* | *Company that produces antibodies* | *Dilution* |
| β-TUBULIN III | Sigma-Aldrich | 1:1000 | Alexa Fluor 488 and 546 | Thermo Fisher Scientific | 1:1000 |
| DCX | Cell Signaling Technology | 1:500 |  |  |  |
| MAP-2 | Sigma-Aldrich | 1:500 |  |  |  |
| NF200 | Sigma-Aldrich | 1:200 |  |  |  |
| Ki67 | Novocastra | 1:500 |  |  |  |
