# Supplementary material for: Mitochondrial biogenesis and neural differentiation of human iPSC is modulated by idebenone in a developmental stage-dependent manner
Source: Biogerontology. 2017 Jun 22;18(4):665–77. doi: 10.1007/s10522-017-9718-4 (PMC5514205; doi:10.1007/s10522-017-9718-4)
Supplement: Supplementary file 2 — Supplementary material 2 (DOCX 17 kb) [file 10522_2017_9718_MOESM2_ESM.docx]

Suppl.Tab.2. Primers used for RT-qPCR

| *Primers* | *Genbank number* | *Primers sequence* | *Amplicon length* |
| --- | --- | --- | --- |
| *POU5F1 F* | *NM_002701.5* | *GAGAGGGGTTGAGTAGTCCCTT* | *100* |
| *POU5F1 R* |  | *CGAAATCCGAAGCCAGGTGTC* |  |
| *SOX2 F* | *NM_003106.3* | *CGGAAAACCAAGACGCTCAT* | *140* |
| *SOX2 R* |  | *TAACTGTCCATGCGCTGGTT* |  |
| *NANOG F* | *NM_024865.3* | *AATAACCTTGGCTGCCGTCT* | *150* |
| *NANOG R* |  | *AGCCTCCCAATCCCAAACAAT* |  |
| *MAP2 F* | *NM_002374.3* | *TGCCTCAGAACAGACTGTCAC* | *101* |
| *MAP2 R* |  | *AAGGCTCAGCTGTAGAGGGA* |  |
| *GFAP F* | *NM_002055.4* | *GTGAAGACCGTGGAGATGCG* | *76* |
| *GFAP R* |  | *TGCCTCACATCACATCCTTGT* |  |
| *NES F* | *NM_006617.1* | *CCCCGTCGGTCTCTTTTCTC* | *96* |
| *NES R* |  | *TCGTCTGACCCACTGAGGAT* |  |
| *NEUROD1 F* | *NM_002500.4* | *ATCTTGCACAGGGAGTCACC* | *90* |
| *NEUROD1 R* |  | *TACTGCCGTCCAGTCCCATA* |  |
| *NEFL F* | *NM_006158.4* | *AGCGTGGGAAGCATAACCAG* | *80* |
| *NEFL R* |  | *CTGGTCTGTAAACCGCCGTA* |  |
| *TUBB3 F* | *NM_006086.3* | *CAACCAGATCGGGGCCAAGTT* | *146* |
| *TUBB3 R* |  | *GAGGCACGTACTTGTGAGAAGA* |  |
| *NRF1 F* | *NM_001001928.2* | *CAGCCGCTCTGAGAACTTCAT* | *148* |
| *NRF1 R* |  | *GTCTTCATCAGCACTCAGCATACTA* |  |
| *TFAM F* | *NM_005011.4* | *TGAAAGATTCCAAGAAGCTAAGGGT* | *132* |
| *TFAM R* |  | *TAACGAGTTTCGTCCTCTTTAGCAT* |  |
| *PPARGC1A F* | *NM_003201.2* | *TAGTAAGACAGGTGCCTTCAGTTC* | *174* |
| *PPARGC1A R* |  | *CTCGATGTCACTCCATACAGACTC* |  |
| *ACTB F* | *NM_001101.3* | *GCTCACCATGGATGATGATATCGC* | *169* |
| *ACTB R* |  | *CACATAGGAATCCTTCTGACCCAT* |  |
| *GAPDH F* | *NM_002046.5* | *GTTCGACAGTCAGCCGCATC* | *90* |
| *GAPDH R* |  | *TCCGTTGACTCCGACCTTCA* |  |
| *HPRT1 F* | *NM_000194.2* | *AGGCGAACCTCTCGGCTTTC* | *166* |
| *HPRT1 R* |  | *CTGGTTCATCATCACTAATCACGAC* |  |
| *TUBB3 F* | *NM_006086.3* | *CAACCAGATCGGGGCCAAGTT* | *146* |
| *TUBB3 R* |  | *GAGGCACGTACTTGTGAGAAGA* |  |
| *EID2 F* | *NM_153232.3* | *GGCATCGCTCTGTCCAGTTA* | *74* |
| *EID2 R* |  | *GCTTGGACATCTCAGACCGT* |  |
| *CAPN10 F* | *NM_023083.3* | *TCTCACCGGGCTACTACCTG* | *86* |
| *CAPN10 R* |  | *CCCGGTAGAGAAGACTCGGA* |  |
| *RABEP2 F* | *NM_024816.2* | *AGGAAGGGGCAAATGGTGAG* | *96* |
| *RABEP2 R* |  | *CAGCCTTCATGGTTTCCATTTCTG* |  |
| *ZNF324B F* | *NM_207395.2* | *CATTGGAAGGACAAACCTAGGATGATG* | *164* |
| *ZNF324B R* |  | *CTTATCTGCTCCAAAGCTATCACTGTC* |  |
| *NAT1 F* | *NM_001160170.3* | *TGGTTGCCGGCTGAAATAAC* | *93* |
| *NAT1 R* |  | *TCTGTCTAGGCCAGTCTCCT* |  |
| *TBP F* | *NM_003194.4* | *GCAAGGGTTTCTGGTTTGCC* | *80* |
| *TBP R* |  | *CAAGCCCTGAGCGTAAGGTG* |  |
| *PHB F* | *NM_001281496.1* | *TGGAAGCAGGTGAGAATGGAG* | *76* |
| *PHB R* |  | *ATCATGGAGCAGAGGAGGACT* |  |
| *UBC F* | *NM_021009.6* | *ACGGGACTTGGGTGACTCTA* | *82* |
| *UBC R* |  | *ATCGCCGAGAAGGGACTACT* |  |
| *CCNG1 F* | *NM_004060.3* | *GCCTCTCGGATCTGATATCGT* | *138* |
| *CCNG1 R* |  | *CATTCAGCTGGTGTAGCAGT* |  |
| *MYC F* | *NM_002467.4* | *CCCTCCACTCGGAAGGACTA* | *96* |
| *MYC R* |  | *GCTGGTGCATTTTCGGTTGT* |  |
| *EEF1A1 F* | *NM_001402.5* | *TGTTCCTTTGGTCAACACCGA* | *122* |
| *EEF1A1 R* |  | *ACAACCCTATTCTCCACCCA* |  |
| *RPLP0 F* | *NM_001002.3* | *CCTCGTGGAAGTGACATCGT* | *76* |
| *RPLP0 R* |  | *CTGTCTTCCCTGGGCATCAC* |  |

*F- forward; R-reverse*
