# Supplementary material for: Mitochondrial biogenesis and neural differentiation of human iPSC is modulated by idebenone in a developmental stage-dependent manner
Source: Biogerontology. 2017 Jun 22;18(4):665–77. doi: 10.1007/s10522-017-9718-4 (PMC5514205; doi:10.1007/s10522-017-9718-4)
Supplement: Supplementary file 3 — Supplementary material 3 (DOCX 14 kb) [file 10522_2017_9718_MOESM3_ESM.docx]

**Suppl.Tab.3.** Primers used for qPCR

| *Gene*  *symbol* | *Genbank*  *number* | *Primers sequence* | *Amplicon length (bp)* |
| --- | --- | --- | --- |
| *SERPINA1 F* | NM_000295.4 | CAGTGAATAAATGAGGCGTACATCC | 89 |
| *SERPINA1 R* |  | GACTGTTTCTCATGCCTCTGGAAAG |  |
| *SLCO2B1 F* | NM_007256.4 | CCTGATGCCTAGGTTTCTTTTCTTG | 85 |
| *SLCO2B1 R* |  | GGTCATCTGCCTACCCTAGAAC |  |
| *mt-ND1 F* | NC_012920.1 | TACGGGCTACTACAACCCTTC | 77 |
| *mt-ND1 R* |  | ATGGTAGATGTGGCGGGTTT |  |
| *mt-ND5 F* | NC_011137.1 | CATTACTAACAACATTTCCCCCGC | 70 |
| *mt-ND5 R* |  | GGCTGTGAGTTTTAGGTAGAGGG |  |

*F- forward; R-reverse*
